# *PTEN* Gene and Autism: Genetic Underpinnings and Neurodevelopmental Impacts

**DOI:** 10.3390/genes16091061

**Published:** 2025-09-09

**Authors:** Ann C. Genovese, Merlin G. Butler

**Affiliations:** Department of Psychiatry and Behavioral Sciences, University of Kansas Medical Center, Kansas City, KS 66160, USA; mbutler4@kumc.edu

**Keywords:** *PTEN* gene, genetic functions and mechanisms, protein interactions, autism, ASD, neurodevelopment, macrocephaly, abnormal CSF dynamics

## Abstract

**Background/Objectives**: Twin and family studies suggest that 90% of the risk for autism spectrum disorder (ASD) is due to genetic factors, with 800 genes recognized as playing a role. An important gene is phosphatase and tensin homolog (*PTEN*), which plays a significant role in cancer as a tumor suppressor best known for causing overgrowth and *PTEN* hamartoma tumor syndromes (PHTS). Less well known are *PTEN* germline mutations with adverse neurodevelopmental impacts of macrocephaly, intellectual disability, and ASD, as well as other behavioral and psychiatric disturbances. There remains a limited understanding of whether these gene variants are associated with differing manifestations of *PTEN*-associated neurodevelopmental disorders. **Methods**: This review utilized comprehensive literature searches such as PubMed, OMIM, and Gene Reviews with keywords of *PTEN*, genetic factors, autism, and human studies and by searching genomic-protein functional networks with STRING computer-based programs for functional and genetic mechanisms. **Results**: This review explored the genetic underpinnings of *PTEN* gene variants causing altered interactive proteins and their mechanisms, biological processes, molecular functions, pathways, and disease–gene associations. We characterized specific gene–gene or protein–protein interactions and their functions relating to neurodevelopment, psychiatric disorders, and ASD that were found to be increased with *PTEN* gene variants. **Conclusions**: *PTEN* gene defects are among the most recognized genetic causes of ASD. *PTEN* gene variants and altered protein interactions and mechanisms described in our study are associated with an increased risk for tissue and organ overgrowth, macrocephaly, and distinct brain anomalies, specifically newly identified abnormal CSF dynamics. These genetic underpinnings and impacts on neurodevelopment are discussed. The genetic and protein findings identified may offer clues to effective treatment interventions, particularly when instituted at a young age, to improve long-term outcomes.

## 1. Introduction

Classic autism is characterized by changes in behavior and developmental disturbances by three years of age due to heterogeneous conditions grouped collectively as autism spectrum disorder (ASD) [1]. Significant impairments in both verbal and non-verbal communication and social interactions are recognized, along with restricted repetitive behaviors, including interests and physical activity [2,3]. Lack of eye contact, sleep disturbances, and tactile defensiveness are common from an early age [4,5,6]. ASD affects nearly 2% of children in the general U.S. population, with a 4:1 male to female ratio, usually without growth or congenital anomalies [7,8]. About 25 percent of children with autism will carry a diagnosis by 2–3 years of age, and 60 percent diagnosed in early childhood will have intellectual disabilities [9,10]. When comparing health disorders with the involvement of the central nervous system, autism ranks higher in frequency than epilepsy, dementia, and Parkinson disease [11,12].

Autism also occurs more commonly than congenital malformations in the general population, with dysmorphic features seen in about 25% of children with autism. Dozens of disorders, mostly of genetic origin, are known for having autism as a recognized finding, such as Fragile X, Williams, Prader–Willi, DiGeorge, and Down syndromes [13]. These syndromic autism disorders represent about 10% of all individuals with ASD and are often associated with congenital malformations or findings characteristic of genetic syndromes. There is a complex interplay between environmental factors and inheritance, with over 800 genes known or clinically involved in autism [14]. Twin and family studies suggest a 90 percent risk for developing autism related to a wide range of causes and candidate genes identified by different means, including chromosome abnormalities and single-gene defects. Chromosomal microarrays identifying small cytogenetic defects are the first tier of genetic testing for patients with ASD to detect submicroscopic deletions or duplications containing genes that play a role and are found in more than 20 percent of affected patients [15].

Identification of causative gene variants as seen in phosphatase and tensin homolog (*PTEN*) is an important source of information to guide treatment, surveillance, and counseling to manage comorbidities and health-related risks, such as seizures, developmental regression, and cancer. Microcephaly is seen in about 10 percent of children with autism, but macrocephaly is reported in 20 to 25 percent of those with *PTEN*-ASD. Hence, those with autism and extreme macrocephaly are at a greater risk of having a *PTEN* tumor suppressor gene defect [16].

*PTEN* gene variants are known to cause overgrowth of tissues and organs and *PTEN* hamartoma tumor syndromes (PHTSs) [17,18]. PHTS refers to a spectrum of disorders commonly due to mutations of *PTEN* with variability in presentation and a range of germline mutations, with clinical findings including colon cancer, esophageal glycogenic acanthosis, penile macules, renal cell carcinoma, testicular lipomatosis, vascular anomalies, and autism spectrum disorders [17]. Questions have been raised as to whether *PTEN* gene variants and specific altered exons may vary between those affected with enlarged head size (macrocephaly) and autism compared with those with *PTEN* gene variants and malignancy.

Mutations in the *PTEN* gene are now considered one of the most important genetic causes of ASD, with other neurodevelopmental findings including elevated risks of macrocephaly and intellectual disability. The focus of this report was to describe the *PTEN* genetic underpinnings, protein interactions, biological processes, pathways, molecular functions, and mechanisms, along with associated diseases, identified using STRING and other searchable computer-based programs and databases. Furthermore, associated macrocephaly and brain anomalies, specifically the recently identified abnormal CSF (cerebral spinal fluid) dynamics of the *PTEN* gene and its encoded protein, will be discussed with regards to their impact on neurodevelopment and autism.

## 2. Materials and Methods

A comprehensive search was conducted utilizing published literature and interactive computer-based programs and databases by focusing on *PTEN*, genetic factors, and autism and human studies (excluding non-human data) using PUBMED (https://pubmed.ncbi.nlm.nih.gov). We extensively utilized the searchable STRING (https://string-db.org) computer program for the study of genomic-protein databases and identified significant protein–protein interactions. This computer-based program and database information will be collected and analyzed from literature to further characterize and predict protein–protein associations, functional mechanisms and their protein networks with top biological processes, molecular functions, cellular components, pathways, and disease–gene associations related to the *PTEN* gene and its encoded protein. The STRING analysis provides a *count in network* category (e.g., biological processes) and will indicate how many proteins in the network are annotated with a particular term and how many proteins in total have this term assigned to this variable per category. *Strength* utilizes a log10 (observed/expected) measure to describe how large the enrichment effect is, based on the ratio between the number of proteins in the network that are annotated with a term and the number of proteins expected to be annotated with this term in a random network of the same size. *Signal* is defined as a weighted harmonic mean between the observed/expected ratio and -log (FDR or false discovery rate). Lastly, this computer program will calculate the *FDR*, which is a statistical measure that examines the significance of enrichment shown as *p*-values and corrected for multiple testing within each category. We searched ‘*PTEN*’ using this program and other computer-based analytical programs for humans (*Homo sapiens*) only during the month and year (July 2025).

Other computer-based searchable sources were also used to provide useful information for human disorders, genes, and protein descriptions, with functions such as the catalog of human genetic disorders from Online Mendelian Inheritance in Man (OMIM) (https://www.omim.org); UniProt (www.uniport.org), a searchable database of protein sequences and functional information; and Ensembl (www.ensembl.org), a genome browser for vertebrate genomes. Other informative genetic sources included GENECARDS (www.genecards.org) and GENEREVIEWS (www.genereviews.org).

## 3. Results

**Figure 1:**
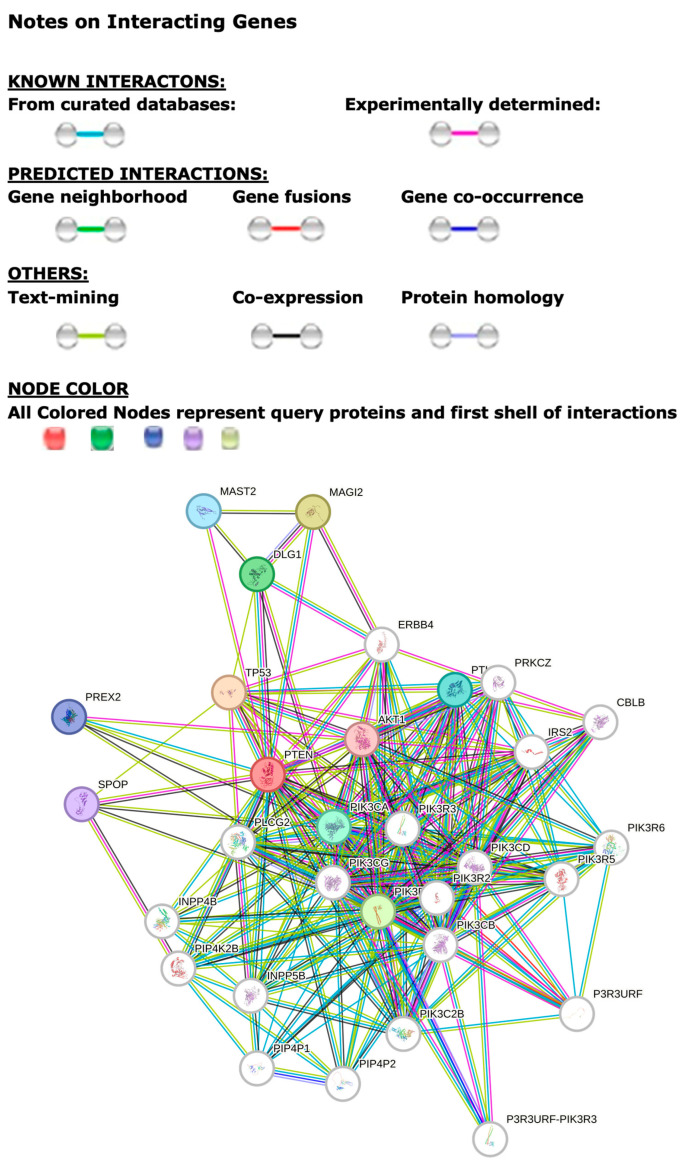
STRING protein–protein interactive network for the *PTEN* gene showing functional involvement for 30 associated protein nodes, along with 227 edges that represent protein–protein interactions associated with specific or meaningful information related to such proteins that contribute jointly to a shared function. Nodes from the protein networks represent proteins that have splice isoforms or post-translational modifications that collapse into each node for all protein forms produced by a single protein-coding gene, including *PTEN*.

The STRING computer-based genomic program and database showed encoded protein–protein associations for the *PTEN* gene. The program identifies biological processes, cellular components, molecular functions, KEGG and Reactome pathways, and disease–gene associations with the top 30 associated or interactive protein nodes for *PTEN*. All interactive proteins with this gene and other encoded proteins included protein isoforms and 227 edges, which indicate both direct and predicted functional and physical protein–protein associations (see Figure 1).

The *PTEN* tumor suppressor gene and its encoded protein acts as a dual-specificity protein phosphatase by dephosphorylating tyrosine-, serine-, and threonine-phosphorylated proteins. As a lipid phosphatase, it removes phosphate in the D3 position of the inositol ring from phosphatidylinositol 3,4,5-trisphosphate, phosphatidylinositol 3,4-diphosphate, phosphatidylinositol 3-phosphate, and inositol 1,3,4,5-tetrakisphosphate with order of substrate preference for in vitro Ptdins, critical for tumor suppressor activity. The STRING-predicted functions of the 30 predicted functions, protein symbols, and descriptions of top significantly associated proteins for *PTEN* are shown in Table 1.

The predicted top functional enrichments identified in our study are shown in Table 2, related to the *PTEN* gene and its encoded protein, biological processes, molecular functions, cellular components with dedicated combined pathways, and their disease–gene associations. The strengths of each relationship, role, and importance are listed within the functional protein network categories.

The *PTEN* gene is found on the chromosome 10q23.31 band and acts as a dual-specificity protein phosphatase. It is ubiquitously expressed as a tumor suppressor that antagonizes PI3K signaling pathways and negatively regulates other pathways, such as MAPK (mitogen-activated protein kinase 1), via phosphatase activity involving phosphatidylinositol processing [19]. We further characterized its role using the searchable STRING computer-based program, technology, and databases, as shown in Table 2.

Initially, phosphatidylinositol or inositol phospholipid was discovered in bacteria and later in eukaryotes and was limited to the endoplasmic reticulum. It serves as a signaling molecule for multiple functions impacting growth and cell regulation, consisting of different isomers as a lipid containing a phosphate group, two fatty acids, and one inositol sugar-related molecule. These isomers have different functions, such as taste sensory, regulation of phosphate levels, mRNA export and translation, metabolic flux, insulin signaling, and involvement in embryo development and stress response [20,21]. Of the 30 network protein nodes identified in Table 1, at least 18 of the 30 proteins are predominantly phosphatidylinositols or related-complex proteins acting in phosphorylation and/or activation of signaling cascades or related mechanisms, which, in turn, are important for the regulation of cellular processes, proliferation, differentiation, and cell death.

## 4. Discussion

### 4.1. Genetic Underpinnings and Mechanisms of PTEN

Although hundreds of genes are recognized to play a role in ASD, identification of specific genetic etiologies remains an area of active investigation. Individuals with overgrowth features and hamartomas are occasionally found to have Proteus or Cowden syndrome, a rare autosomal-dominant disorder with a high risk of cancer. These individuals may have a *PTEN* gene defect when studied. More recently, they have been reported with neurobehavioral findings, including autism, intellectual disability, and macrocephaly [16,22,23]. Mutations or variants of the *PTEN* gene, a tumor suppressor gene, was later recognized to cause *PTEN* hamartoma tumor syndromes, but its roles in behavior and learning problems have been understudied [17,18]. For example, Butler et al. analyzed 18 subjects (ages 3–8 years) with ASD and macrocephaly with head circumferences of 2.5–8 SD above the mean. They found that three males with the largest head circumferences had germline *PTEN* gene mutations or variants [16]. *PTEN* gene variants are now recognized as important causes of ASD, particularly those with macrocephaly and with larger frontal but smaller occipital lobes, as seen in about 20 percent of children with autism [14,23].

Increased cerebral spinal fluid (CSF) leading to ventriculomegaly is recognized in congenital hydrocephalus and is an understudied finding in autism [24]. De novo mutations or variants of the *PTEN* gene are now recognized as frequent causes of congenital hydrocephalus and primary ventriculomegaly. In addition, mice models with *PTEN* gene defects show ventriculomegaly and aqueductal stenosis related to hyperproliferation of periventricular Nkx2.1+ neural progenitor cells (NPCs), with increased CSF production from the hyperplastic choroid plexus. This disruption leads to network dysfunction from increased activity of the altered NPC-derived inhibitory interneurons and protein production that concurrently affects CSF dynamics. It has been noted that both aqueductal stenosis and ASD are more common in males with *PTEN* mutations [24].

Normal *PTEN* function is needed, as it interacts with other important and related genes, with their encoded proteins being involved in early cell growth, cycle, differentiation, and tumor development, with damaging effects on the early developing brain, impacting neural progenitor cells, CSF dynamics, dysregulation, and brain formation [24]. These detrimental derangements impact early brain formation and size by involving the entire neurological system, regulation, and function of neural progenitor cells required for normal nerve cell growth and brain size, proliferation, survival, and differentiation. About 20 percent of patients with sporadic congenital hydrocephalus will show *PTEN* gene variants [25]. Hence, better understanding of the human CSF system and production with transport has the potential to elucidate key aspects of corticogenesis, synaptogenesis, and circuit formation, with a role for *PTEN* needed for neurodevelopment and causing autism when disturbed [24].

*PTEN* is highly expressed in inhibitory NPCs, with their derived cortical interneurons required for neuron development and function in the early embryo [24]. *PTEN* and other genes are associated with neural stem cell (NSC) development and fate regulation, including the *TRIM71* gene, which is involved in regulating the timing of stem cell progression during early development [24,26]. *PTEN* controls alternative splicing of ASD-associated transcripts in primary neurons and is the main antagonist of the phosphatidylinositiol-3-kinase (PI3K)/AKT/mTOR signaling pathway mutated in 10–20% of individuals with ASD presenting with macrocephaly [27,28]. In addition, inflammation is a potential outcome of CSF hypersecretion, causing ventriculomegaly, an area in need of more research [24]. Conversely, ventriculomegaly is the most common antenatally diagnosed brain anomaly identified in humans [29].

*PTEN* encodes a lipid phosphatase that counteracts phosphoinositide 3-kinase by dephosphorylating phosphatidylinositol-(3,4,5)-triphosphate and antagonizes the AKT-mTOR signaling pathway to regulate cell growth, proliferation, and survival [30,31]. Disruptions of *PTEN* are also implicated in overgrowth disorders, cancer, and neurological conditions, including autism, epilepsy, and macrocephaly [32]. Interestingly, mTORC1 (mammalian target of rapamycin complex 1) inhibition via specified NPCs may rescue ventriculomegaly and parenchymal brain interneuronopathy caused by *PTEN* depletion, and, hence, mTORC1 inhibitors (e.g., rampamycin) have been used to treat tuberous sclerosis, a recognized genetic disorder with *TSC1* and *TSC2* gene defects but with autism as a feature and *PTEN* hamartomas [33,34,35]. In addition, Everolimus, an approved drug for partial seizures in children and adults with tuberous sclerosis, has been used with potential success in controlled clinical trials in treating individuals with *PTEN*-associated ASD and neurodevelopmental disorders [36,37].

*PTEN* interacts with TP53 (tumor protein p53), a cellular tumor antigen, and other genes with their encoded proteins, such as MAGI2, AKT1, and PIK3R1, along with other members of the *PIK3* gene family which are involved with protein kinase signaling and binding, with phosphatase activity impacting the cell-to-cell junction and myelin sheaths, as described in Table 1. These disturbances presumably alter the VEGFA and PI3K/AKT pathways, leading to uncontrolled cell growth and malignancies, such as small cell lung and endometrial cancers. The *MAGI2* gene interacts with *PTEN* and generates a scaffold protein at synaptic junctions required for nerve growth factor recruitment, affecting brain development, size, and function. The interactive PI3K signaling and related genes with encoded proteins regulate cell growth and proliferation in multiple tissues, including neurons. Somatic *PIK3CA* and other members of the gene family, along with *mTOR* gene mutations, drive tumorigenesis through increased mTOR signaling to promote brain NPC proliferation and differentiation by phosphorylation.

Multi-omics refers to the integration and analysis of data from multiple “omics” fields, such as genomics, transcriptomics, proteomics, and metabolomics, to gain a better understanding of biological systems. It involves combining data from different “omes” to identify complex mechanisms within cells or tissues, for example, and is used to learn about disease mechanisms and identify potential therapeutic targets. Studies with the use of multi-omics and cells and tissues of specific brain regions collected for research from those with autism (syndromic and non-syndromic) should be undertaken to better understand the role and causation, potentially leading to therapeutic applications [38].

Gabrielli et al. [39] generated a combined gene list for both autism (792 associated genes) and malignancy (3500 associated genes), with 138 autism genes (17% of all identified autism genes) recognized as cancer genes. They used the GeneAnalytics computer-based program and analyzed genes for diseases, pathways, phenotypes, biological processes, and molecular functions. The analysis included matched disease entities with genetic- and protein-related factors to implicate the shared pathology of autism and cancer genes. They identified seven significantly associated diseases, including colorectal and breast cancers, as the top diseases when analyzing the 138 shared autism- and cancer-related genes. They found that 54% and 32%, respectively, involving ERK (extra-cellular signal-regulated kinase) and GPCR (G-protein coupled receptor) are related to major cell-signaling and pathways. Additionally, metabolic factors and biological processes involving protein binding, gene transcription, and cell growth were also found, raising concerns about cancer risk in those with autism, specifically with the involvement of one or more autism-related genes that are associated with cancer.

To determine whether gene variants clustered in specific exons of the *PTEN* gene vary between those with macrocephaly and ASD, compared with those with *PTEN* gene variants and malignancy without autism, we examined literature. The *PTEN* gene consists of nine exons, and its encoded protein consists of 403 amino acids (www.genecards.org). We found two literature sources to assist, whereby *PTEN* gene mutations were systematically reviewed for clinical implications, and the location of the gene variant identified in those with ASD and increased head size or macrocephaly [40] or those with malignancy alone as having multiple tumor types, such as endometrial, ovarian, breast, pancreatic, and glioma [41]. A total of 481 *PTEN* gene variants involving multiple tumor types were found in all nine exons, with exon 5 accounting for 49 percent of the variants, followed by exon 8 with 15 percent and exon 7 with 11 percent. Similarly, Zahedi et al. [40] reported gene mutations in patients with macrocephaly and classic autism and identified 29 patients with *PTEN* variants and exon locations. Exon 5 was most commonly involved with 9 of the 29 defects (31 percent), followed by exon 6 with 6 defects (21 percent) and exon 1 with 5 defects (17 percent). Hence, these preliminary data show, in either cancer or ASD, that exon 5 was the most common exon with defects when comparing both groups of patients.

In summary, our STRING computer-based analysis reviewed biological processes, molecular functions, cellular components, pathways, and disease–gene associations for the *PTEN* gene and its encoded protein when disturbed and their interactions. The function of the interactive proteins identified are listed in Table 1 and Table 2, showing proteins that induce growth arrest with negative regulation of cell division (e.g., TP53). Generated scaffold molecules are required at synaptic junctions and for assembling neurotransmitter receptors, nerve growth factor recruitment, cell adhesion proteins, channels, and signaling molecules (e.g., MAGI2, DLG1), along with ubiquitin ligase and proteosomal degradation, required in neurodevelopment, head size, and craniofacial features (e.g., SPOP). Megalocephaly, abnormal axon growth, neuronal branching, or abnormal synapse formation results in brain malformations and altered CSF dynamics, as reported in ASD.

Regulation of cell migration is required for growth, adhesion, spreading, cytoskeleton development, and microtubule filaments needed for early embryo development (e.g., AKT2; PTK2; MAST2). Cellular-level phosphorylation and activation of signaling cascades involving *PTEN* and interactive proteins are required for cell growth survival, proliferation, motility, and morphology. This requires glucose uptake and energy sources for normal cellular response to stress and tumor growth, syndrome development, vascular changes, and megalocephaly (e.g., PIK3R1; PIK3CA; PIK3CG; PIK3CD; PIK3CB; PIK3R3; PIK3R2; PIK3R5; PIK3R6). *PTEN* disturbances and interactions with other related proteins, as described in our study, lead to neurodevelopmental and brain deficits in migration and lamination, hypertrophy, neurite outgrowth, and spine formation with connectivity [42].

The majority of the top 30 interactive proteins with *PTEN* identified using the STRING computer program are also directly involved with phosphatidylinositol 3-kinase catalytic or regulatory subunits and phosphatidylinositol 4,5-bisphosphate subtypes. These protein interactions include PI3K/AKT/phosphate and tensin homolog (*PTEN*), noted to play early roles in cellular phosphorylation and activation of brain growth with survival, development, and differentiation related to ASD. These proteins are associated with autoinflammation, immunodeficiency, and glucose uptake, serving multiple functions, including being a second messenger for normal growth and regulation, leading to overgrowth, cancer, and ASD when altered by *PTEN* gene defects [42].

### 4.2. Neurodevelopmental Impacts of PTEN Mutations

*PTEN* germline mutations have adverse neurodevelopmental impacts, including elevated risks of macrocephaly, intellectual disability, and ASD. *PTEN* is a crucial regulator of brain development and function, impacting neurogenesis, synaptogenesis, synaptic connectivity, myelination, plasticity, and neuronal survival, as noted in our study and elsewhere [43]. The most common clinical finding in humans with mutated *PTEN* is macrocephaly, with the increased head circumference caused by enlargement of the cerebellum, ventricles, and white matter, with the involvement of CSF dynamics [24,44,45]. The estimated prevalence of macrocephaly (defined as >2 SD above the mean) in *PTEN* mutations ranges from 25 to 100% [46,47]. Pathogenic *PTEN* mutations have been identified in about 2% of all individuals with ASD and about 20% of cases with ASD and macrocephaly [48,49,50].

Autism spectrum disorder is associated with disruptions in brain development that lead to aberrant patterns of brain connectivity, affecting how different brain regions communicate and interact. The loss of *PTEN* function in the CNS leads to aberrant brain connectivity, increasing excitatory synaptic connectivity and thus contributing to an imbalance between neuronal excitation and inhibition [45]. ASD is highly heritable and may result from different genetic anomalies, including chromosomal copy number variation, single gene mutations, epigenetic changes, and complex inheritance [51,52]. Multiple discoveries of de novo *PTEN* mutations have confirmed their role as a risk gene for ASD [53]. Despite this knowledge, only 10–20% of ASD cases previously identified with an etiology, but with newer studies using advanced genomic technology, higher causative rates approach 50% [23,54,55].

The manifestation of ASD in *PTEN* gene mutations is often described as syndromic autism [56]. Syndromic autism refers to ASD associated with clinically defined genetic syndromes, with a defined pattern of somatic abnormalities and a distinct neurobehavioral phenotype. Unlike idiopathic autism, which has no identifiable cause, syndromic autism is associated with specific genetic disorders that often come with additional features beyond those typically associated with ASD [57]. The genetic mutations causing these syndromes significantly increase the risk of ASD, with the specific features of syndromic autism varying between different genetic disorders [58]. Syndromic autism has been described in numerous genetic disorders, including Fragile X syndrome, tuberous sclerosis complex, Phelan–McDermid syndrome, Prader–Willi syndrome, Angelman syndrome, DiGeorge syndrome, phenylketonuria, Down syndrome, Rett syndrome, Williams syndrome, Burnside–Butler syndrome, and Cornelia de Lange syndrome [13].

A meta-analysis estimated the prevalence of ASD or ASD characteristics in *PTEN* mutations at approximately 25%, with an increased associated risk of co-occurring intellectual ability and adaptive functioning in PTEN-ASD, compared to PTEN-no ASD [59]. It should also be noted, however, that cognitive abilities have been reported to vary greatly, with some individuals’ IQs being significantly above the population mean [60]. In contrast to idiopathic ASD, so-called PTEN-ASD appears to have a dissimilar neurobehavior and clinical phenotype. A distinct neuropsychological profile has been described with mutations in *PTEN* which indicate greater frontal lobe dysfunction (i.e., impairments in attention, impulse control, reaction time, processing speed, and motor coordination), lower clinical ratings of autism severity, and lower sensory responsiveness [60].

Eng and others found in a large sample of individuals with *PTEN* gene defects that those with ASD co-occurring with other developmental disorders had an overall increased burden of copy number variants (CNVs) [22,61]. Specifically, 10.0% of patients with ASD or NDD had identifiable neurodevelopmental disorder-associated CNVs, compared to 2.6% of patients without ASD or NDD and 1.7% of patients with cancer, suggesting that CNVs may act as genomic modifiers that influence the clinical presentation in individuals with *PTEN* mutations, shifting the risk toward ASD or other NDDs versus cancer [61].

### 4.3. Neurobehavioral and Neuropsychiatric Impacts of PTEN Mutations

The impacts of *PTEN* germline mutations include elevated risks of neurobehavioral and neuropsychiatric disorders, the specific manifestations of which are highly variable [45,49,62]. Increased rates of behavioral and psychiatric disorders which have been reported with *PTEN* mutations include attention deficit hyperactivity disorder (ADHD), disruptive behavior disorder, oppositional defiance disorder, irritability, impulsive aggression, generalized anxiety disorder, depressive disorders, bipolar disorder, and obsessive–compulsive disorder [63]. It is believed that characteristic focal abnormalities in white matter that are known to occur in *PTEN* mutations contribute to the risk for co-occurring psychiatric diagnoses with *PTEN* mutations [64,65].

Elevated anxiety symptoms have been reported in up to 50% of individuals with *PTEN* mutations, with the notable exception of social anxiety, with many of these children described as having strong social motivation. Higher levels of anxiety are moderately correlated with sensory features, supporting previous research demonstrating the relationship between sensory sensitivity and anxiety in children with ASD. Atypical sensory processing has also been associated with self-injurious behaviors in *PTEN* mutations, as well as autism severity, lower adaptive ability, and higher anxiety levels [66].

*PTEN* mutations occurring with ASD have significantly higher levels of anxiety and mood disorders than *PTEN* without ASD but lower levels of irritability and emotional dysregulation. In fact, clinical observations have described these individuals as being generally happier and more adaptable to changes in routine and environment, compared to those with idiopathic autism [48]. When *PTEN* mutations are associated with ASD and macrocephaly, externalizing behavior disorders with attention deficits and aggression are more commonly reported [44,48,67].

There is significant growing evidence of the frequent association between ASD and irritability, aggression, self-injurious behaviors, ADHD, anxiety, obsessive–compulsive disorder, gender dysphoria, mood disorders, suicidality, substance use disorders, catatonia, psychosis, and schizophrenia spectrum disorders [68]. Diagnostic challenges occur as the result of symptom overlapping of these disorders with core characteristics of ASD. Elevated risks of behavioral and psychiatric disorders are associated with both *PTEN* mutations and ASD, suggesting the risk potentially occurs in an additive manner, indicating more research is needed.

## 5. Conclusions

*PTEN* mutations or gene variants are among the most recognized genetic causes of ASD. *PTEN* gene variants are associated with an increased risk for tissue and organ overgrowth; macrocephaly with distinct brain findings, including abnormal CSF dynamics; and neurodevelopmental–psychiatric disorders. There is growing evidence that effective treatment interventions, particularly when instituted starting at a young age, can improve long-term outcomes. Identification of genetic disorders and specific gene defects requires chromosomal microarrays and/or next-generation DNA sequencing, particularly in those patients presenting with neurodevelopment–psychiatric disorders. *PTEN* gene defects or other abnormal gene findings may be diagnostic. Those with overgrowth, macrocephaly, and abnormal brain findings, such as errors in CSF dynamics, are at a greater risk of having an involvement of the *PTEN* gene.

*PTEN* genomic-protein interactions, shared functions, pathways, and molecular mechanisms, as described in our study, showed involvement of signaling pathways, metabolic factors, and biological processes, including protein binding, gene transcription, and cell growth. Interactions between PI3K/AKT/phosphate and tensin homolog (*PTEN*) were found to play early roles in cellular phosphorylation and activation with brain growth and survival but were related to overgrowth, differentiation, and development in ASD. Proteins were also associated with autoinflammation, immunodeficiency, and glucose uptake, requiring more research. *PTEN* germline mutations adversely impact neurogenesis, synaptic connectivity, myelination, survival, plasticity, head size (CSF dynamics), intellectual disability, and ASD. This acquired information will help identify priorities for future laboratory and clinical research to assist patients and families in gaining timely access to effective evidence-based treatment and genetic counseling based on their genetic cause.

## Figures and Tables

**Table 1:** Protein symbols and related descriptions of the top 30 associated *PTEN* proteins *.

Protein Symbol	Description
TP53	Cellular tumor antigen p53 acts as a tumor suppressor in many tumor types and induces growth arrest or apoptosis, depending on physiological circumstances and cell type, with negative regulation of cell division.
MAGI2	It generates scaffold molecules at synaptic junctions and assembles neurotransmitter receptors, along with proteins, for cell adhesion required for nerve growth factor recruitment.
MAST2	It is linked to the dystrophin/utrophin network and involved with microtubule filaments using syntrophins that affect muscles and spermatid maturation. It is a representative member of the protein kinase superfamily.
SPOP	It functions within the ubiquitin ligase complex pathway and is required for proteosomal degradation of targeted proteins. It is also involved in cell cycle regulation that plays a role in global development, behavior, head size, and craniofacial features.
DLG1	Disks large homolog 1 is an essential multidomain scaffolding protein required for normal development and function, including regulation of cardiac myocytes.
PREX2	It functions as a RAC1 guanine nucleotide exchange factor (GEF), which activates Rac proteins through exchanging bound GTP for free GTP. This encoded protein plays a role in the important mediation of Rac signaling, which is downstream to both G protein-coupled receptors and phosphoinositide 3-kinase.
AKT1	This encoded protein is one of three closely related serine/threonine kinases (AKT1, AK2, and AKT3) that generates specific inositol lipids that are implicated in the regulation of cell growth, survival, proliferation, and differentiation, along with cytoskeletal changes impacting cancer and cell overgrowth with the involvement of glucose transportation.
PIK3R1	Phosphatidylinositol 3-kinase is a lipid kinase that phosphorylates the inositol rig of phosphatidylinositol and related compounds at the 3-prime position thought to serve as second messengers in growth signaling pathways.
PIK3R3	This encoded protein binds phosphorylated Tyr kinases as an adapter that mediates an association with the p110 catalytic unit to the plasma membranes, which is necessary for the insulin-stimulated increase in glucose uptake, along with glycogen synthesis. It plays a vital role in the signaling of fibroblast growth factor receptors and for modulation of cellular response to stress.
PIK3CG	Phosphatidylinositol 3-kinase, catalytic, gamma is thought to play a role in autoinflammation and immunodeficiency
PIK3CA	The encoded protein generates cellular phosphorylation for the activation of signaling cascades for cellular growth, proliferation, survival, motility, and morphology-affecting diseases, such as breast, ovary, lung, liver, colorectal, and gastric cancer. Other disorders impacted by this protein when disturbed include Cowden syndrome, cerebral cavernous malformations, overgrowth, body and facial asymmetry, macrocephaly, skin and vascular malformations, lipomatous tumors, megalencephaly, CLOVE, and CLAPO syndromes.
PIK3CB	Phosphatidylinositol 3-kinase, catalytic, 110-kd, beta is implicated in signaling pathways regulating cell growth in response to mitogenic stimuli.
PIK3CD	Phosphatidylinositol 3-kinase, catalytic, 110-kd, delta displays a broad phosphoinositide lipid substrate specificity.
PIK3R5	Phosphatidylinositol 3-kinase, regulatory subunit 5 is involved with the recruitment and activation of cytosolic effectors involved in proliferation, survival, or chemotaxis.
PIK3R6	Phosphatidylinositol 3-kinase, regulatory subunit 6 is involved with phosphoinositide 3-kinase gamma and cellular function and regulation.
P3R3URF	Readthrough transcript encodes a fusion protein that shares sequence identity with the PIK3R3 protein.
INPP4B	Inositol polyphosphate-4-phosphatase, type II, 105-kD is a Mg(2+) independent phosphatase that catalyzes the hydrolysis of the 4-position phosphate from phosphatidylinositol 3,4-biphosphate, inositol 1,3,4-triphosphate, and inositol 3, 4-bisphosphate.
PIP4P1	Phosphatidylinositol 4,5-bisphosphate 4- phosphatase, type I catalyzes the degradation of phosphatidylinositol 4,5-bisphosphate.
PIP4P2	Phosphatidylinositol 4,5-bisphosphate 4- phosphatase, type II catalyzes the degradation of phosphatidylinositol 4,5-bisphosphate.
PTK2	This encoded protein is required for normal regulation of cell migration, adhesion, and spreading, with reorganization of cytoskeleton needed for early embryo and placenta development. It is also required to regulate embryonic angiogenesis, cardiomyocyte migration, and for proliferation of normal axon growth with neuronal cell migration, branching, and synapse formation.
PIP4K2B	Phosphatidylinositol 5-phosphate 4-kinase, beta is required for the final step in the synthesis of the second messenger and phosphorylation of PIP.
PIK3C2B	Phosphatidylinositol 3-kinase, class 2, beta is required to convert phosphoinositides as lipid molecules to crucial roles in diverse cellular functions.
INPP5B	Inositol polyphosphate-5-phosphatase, 75-kD protein functions as an enzyme to regulate calcium signaling by inactivating inositol phosphates located in the cytosol, mitochondria, and cell membranes.
PLCG2	Phospholipase 2, gamma 2 acts as an enzyme to catalyze the hydrolysis of phospholipids and plays a role in autoinflammation and immune dysregulation.
ERBB4	ERB-B4 is a type 1 receptor tyrosine kinase subfamily that includes EGFR and ERBB3.
CBLB	CAS-BR-M murine ecotropic retroviral transforming sequence B is a key regulator of peripheral immune tolerance by limiting T-cell activation and expansion through its E3 ubiquitin ligase activity.
PRKCZ	Protein kinase C, zeta form is involved with secondary messengers that have N-terminal regular domains and C-terminal catalytic domains.
IRS2	Insulin receptor substrate 2 is involved with noninsulin-dependent diabetes mellitus.
PIK3R2	Phosphatidylinositol 3-kinase, regulatory subunit 2 is involved with phosphorylation, serving as a second messenger for growth signaling pathways, leading to megalencephaly and brain malformations when disturbed.
P3R3URF-PIK3R3	Readthrough transcript encodes a fusion protein that shares sequence identity with the PIK3R3 protein and is adjacent to the P3R3URF gene.

* STRING database (www.string-db.org); Online Inheritance in Man (www.omim.org); GeneCards database (www.genecards.org).

**Table 2:** STRING: predicted functions, position within protein networks and their interactions related to the *PTEN* gene and encoded protein *.

Biological Process (Gene Ontology) [Count in Network]	Molecular Function (Gene Ontology) [Count in Network]	Cellular Component (Gene Ontology) [Count in Network]	KEGG Pathway [Count in Network]	Reactome Pathway [Count in Network]	Disease–Gene Association [Count in Network]
Phosphatidylinositol metabolic process [17 of 153 processes]	Phosphatidylinositol 3-kinase activity [7 of 12 functions]	Phosphatidylinositol 3-kinase complex, class 1A [9 of 9 components]	Phosphatidylinositol signaling system [14 of 94 pathways]	Synthesis of PIPs at the plasma membrane [13 of 53 pathways]	Head and neck cancer [4 of 12 associations]
Phosphatidylinositol-3-kinase signaling [11 of 45]	1-phosphatidylinositol-3-kinase activity [6 of 10]	Phosphatidylinositol 3-kinase complex [11 of 30]	ErbB signaling pathway [11 of 81]	PI metabolism [14 of 83]	Proteus syndrome [3 of 3]
Phosphatidylinositol phosphate biosynthetic process [12 of 66]	Phosphatidylinositol phosphate kinase activity [6 of 15]	Phosphatidylinositol 3-kinase complex, class1B [4 of 4]	Glioma [10 of 71]	GPVI-mediated activation cascade [10 of 35]	Cowden syndrome [3 of 10]
Phosphatidylinositol biosynthetic process [14 of 126]	1-phosphatidylinositol -3-kinase regulator activity [6 of 16]	Extrinsic component of membrane [13 of 323]	Inositol phosphate metabolism [10 of 72]	Erythropoietin activates phosphoinositide-3-kianse (PI3K) [7 of 12]	Head and neck squamous cell carcinoma [3 of 11]
Phosphatidylinositol -3-phosphate biosynthetic process [7 of 21]	Phosphatidylinositol -4,5-bisphosphate 3-kinase activity [5 of 7]	Transferase complex [12 of 847]	VEGF signaling pathway [9 of 56]	Signaling by erythropoietin [8 of 25]	Endometrial carcinoma [3 of 9]

* The STRING-predicted function listed with the most significant level based on count in network, strength, signal, and FDR (false discovery rate) is ordered first in each column. For example, the *p*-value for biological processes (phosphatidylinositol metabolic process), it was 1.31 × 10^−23^; for molecular functions (phosphatidylinositol 3-kinase activity), it was 2.8 × 10^−12^; for cellular components (phosphatidylinositol 3-kinase complex, class IA), it was 7.98 × 10^−19^; for KEGG pathways (phosphatidylinositol signaling system), it was 7.46 × 10^−22^; for Reactome pathways (synthesis of PIPs at the plasma membrane), it was 8.38 × 10^−22^; and for disease–gene associations (head and neck cancer), it was 4.11 × 10^−5^. All FDR-derived *p*-values calculated for features in each column or category were significant.

## Data Availability

No available data to share.
